# Comment on “Measuring optic nerve sheath diameter using ultrasonography in patients with idiopathic intracranial hypertension”

**DOI:** 10.1055/s-0043-1768673

**Published:** 2023-06-28

**Authors:** Martina De Luca, Danilo Biondino, Marco Gioia, Aniello La Marca

**Affiliations:** 1 University of Salerno, Department of Medicine, Surgery and Dentistry “Scuola Medica Salernitana”, Baronissi SA, Italy.

Dear Editor,

We are writing to comment on Dağdelen et al.'s study on the measurement of the optic nerve sheath diameter (ONSD) in patients with idiopathic intracranial hypertension. 1

Ocular ultrasonography is considered to be the least invasive diagnostic method to detect intracranial hypertension, even in an emergency setting.

In their paper, the authors utilized the B-scan technique, with the probe placed in the superolateral of the globe, with the upper eyelid closed. The B-scan technique is very sensitive in detecting small optic nerve calcifications, such as in cases of optic nerve drusen, 2 but it is not so reliable for measurements because the optic nerve sheath's appearance is influenced by the B scan gain: with decreased gain, the ONSD appears larger compared to the one acquired with an increased gain (blooming effect). 3 4

Other problems are related to the performance of the examination through closed eyelids, which makes impossible the recognition of gaze position and leads to Bell's phenomenon, with an upward and outward movement of the eye when an attempt to close the eyes is made. The consequence is the acquisition of images of optic nerve in different positions and measurements of sheath diameter stretched differently, thus not comparable.

Moreover, with the eyelids closed, there is a decrease of the image quality score due to the sound attenuation produced by the lids, making the results even more unpredictable.

For this reason, we suggest performing ocular ultrasonography with open eyelids, using methylcellulose and anesthetic drops.

However, the best way to perform the examination is with a standardized A scan. It permits the unequivocal measurement of the ONSD with its hyperreflective spikes from the interface between the arachnoid and subarachnoid fluids, thus making the blooming effect harmless. 5

There are a few other points that, in our opinion, deserve to be discussed.

The case group is meaningfully heterogeneous: in fact, some of the patients (57.4%) were treated with acetazolamide, while the remaining (42.6%) were not.

The authors found a negative correlation between ONSD and age, and a positive correlation between ONSD and body mass index (BMI).

Unfortunately, both case and control groups are significantly different in age and BMI, making the comparison ineffective.

We are afraid that all these problems could make the results of this study non significative.
